# Competitive sgRNA Screen Identifies p38 MAPK as a Druggable Target to Improve HSPC Engraftment

**DOI:** 10.3390/cells9102194

**Published:** 2020-09-29

**Authors:** Denise Klatt, Teng-Cheong Ha, Maximilian Schinke, Anton Selich, Anna Lieske, Julia Dahlke, Michael Morgan, Tobias Maetzig, Axel Schambach

**Affiliations:** 1 Institute of Experimental Hematology, Hannover Medical School, 30625 Hannover, Germany; Denise_Klatt@DFCI.HARVARD.EDU (D.K.); Ha.Tengcheong@mh-hannover.de (T.-C.H.); schinke.maximilian@mh-hannover.de (M.S.); selich.anton@mh-hannover.de (A.S.); lieske.anna@mh-hannover.de (A.L.); dahlke.julia@mh-hannover.de (J.D.); morgan.michael@mh-hannover.de (M.M.); maetzig.tobias@mh-hannover.de (T.M.); 2 REBIRTH Research Center for Translational Regenerative Medicine, Hannover Medical School, 30625 Hannover, Germany; 3 Pediatric Hematology and Oncology, Hannover Medical School, 30625 Hannover, Germany; 4 Division of Hematology/Oncology, Boston Children’s Hospital, Harvard Medical School, Boston, MA 02115, USA

**Keywords:** HSPCs, X-linked chronic granulomatous disease (X-CGD), p38 MAPK, CRISPR-Cas9, bone marrow transplantation, interleukin-1 beta

## Abstract

Previous gene therapy trials for X-linked chronic granulomatous disease (X-CGD) lacked long-term engraftment of corrected hematopoietic stem and progenitor cells (HSPCs). Chronic inflammation and high levels of interleukin-1 beta (IL1B) might have caused aberrant cell cycling in X-CGD HSPCs with a concurrent loss of their long-term repopulating potential. Thus, we performed a targeted CRISPR-Cas9-based sgRNA screen to identify candidate genes that counteract the decreased repopulating capacity of HSPCs during gene therapy. The candidates were validated in a competitive transplantation assay and tested in a disease context using IL1B-challenged or X-CGD HSPCs. The sgRNA screen identified *Mapk14* (*p38*) as a potential target to increase HSPC engraftment. Knockout of *p38* prior to transplantation was sufficient to induce a selective advantage. Inhibition of p38 increased expression of the HSC homing factor CXCR4 and reduced apoptosis and proliferation in HSPCs. For potential clinical translation, treatment of IL1B-challenged or X-CGD HSPCs with a p38 inhibitor led to a 1.5-fold increase of donor cell engraftment. In summary, our findings demonstrate that p38 may serve as a potential druggable target to restore engraftment of HSPCs in the context of X-CGD gene therapy.

## 1. Introduction

Patients with X-linked chronic granulomatous disease (X-CGD) suffer from severe recurrent bacterial and fungal infections [1,2]. The disease is caused by mutations in the NADPH oxidase, which is localized in the phagosomal membrane of granulocytes and macrophages [3]. The main function of the NADPH oxidase is the production of reactive oxygen species, which mediate killing of encountered pathogens via the release and activation of granule proteases [4,5]. Allogeneic hematopoietic stem cell transplantation (HSCT) was previously the only curative treatment option for X-CGD. Despite improvements in conditioning and immunosuppressive regimens, the overall survival after HSCT is about 91% with an event-free survival of 82% [6]. Major risks associated with HSCT are graft failure and acute or chronic graft-versus-host disease. Comorbidities are even higher in the absence of a matched family donor. As many X-CGD patients lack a matched donor, other treatment strategies are required for these patients [7,8]. An alternative treatment option is retroviral gene therapy of hematopoietic stem cells (HSCs), which is tested in clinical trials [9]. Previous trials used LTR-driven gamma-retroviral vectors to deliver the therapeutic transgene and a partial myeloablative conditioning regimen to create space in the bone marrow niche, but resulted in only a short-term clinical benefit [10,11,12]. Despite the clearance of pre-existing, refractory infections in some patients, the trials were hampered by the occurrence of insertional mutagenesis, transgene silencing, and lack of long-term engraftment [13]. To address these limitations, a self-inactivating (SIN) lentiviral vector with a myeloid-specific promoter and a complete myeloablative conditioning is used in a current clinical trial for X-CGD [9]. Preliminary results of this study demonstrated improved and prolonged presence of NADPH oxidase-positive neutrophils as well as polyclonal engraftment of corrected HSCs. Despite these promising findings, one pediatric patient still showed a decline in NADPH oxidase-positive cells over time.

The maintenance of stemness and self-renewal during the ex vivo cultivation and genetic manipulation of isolated HSCs is essential to achieve long-term engraftment after gene therapy [14]. Recent studies demonstrated that chronic inflammation in X-CGD patients resulted in increased levels of pro-inflammatory cytokines in the bone marrow [15]. These pro-inflammatory cytokines include interleukin-1 beta (IL1B) and the chemokines CXCL10, CCL5, and CXCL9. Hyperinflammation and IL1B were identified as main drivers of HSC exhaustion in X-CGD patients by cell cycle entry of quiescent HSCs and a subsequent loss of their long-term repopulating capacity. The loss of HSC quality and quantity most likely contributed to the poor engraftment of gene-corrected cells in X-CGD patients [13,15].

Several hundred genes were identified to play a role in HSC functions [16]. Most genes were essential for the hematopoietic system as their loss of function resulted in a defective hematopoiesis with either a mild (e.g., *Cdkn1a* or *Hif1a*) or severe (e.g., *Hoxa9*) phenotype [17,18,19]. Only a small number of genes anticorrelate with HSC functions, e.g., *Cbl* and *Lnk* [20,21]. Upon competitive transplantation, these knockout cells had a selective advantage and outcompeted wild type cells even in serial transplantations.

In this study, we used CRISPR-Cas9 to knockout several candidate genes and analyzed the effect on the repopulating capacity of hematopoietic stem and progenitor cells (HSPCs) during bone marrow transplantation. Our small sgRNA screen readily identified *p38* as a druggable target to improve the engraftment of healthy and X-CGD-like HSPCs after transplantation.

## 2. Materials and Methods

### 2.1. Mice

B6J.129(Cg)-Gt(ROSA)26Sortm1.1(CAG-cas9*,-EGFP)Fezh/J (Cas9, Jackson Laboratory, Bar Harbor, ME, USA) [22], B6.SJL-PtprcaPep3b/BoyJ (CD45.1) and C57BL/6J (CD45.2) mice were used for competitive transplantation experiments. All mice were bred and maintained in a pathogen-free environment at the animal facility at Hannover Medical School. All animal experiments were performed according to the animal protection law and under control of the Lower Saxony State Office for Consumer Protection and Food Safety (LAVES).

### 2.2. Lentiviral Vectors and Vector Production

CRISPR-Cas9 was used to knockout the candidate genes. For simplicity, we used transgenic mice, which constitutively express the Cas9 (see above), as a cell source. Thus, to introduce a knockout in these cells, the respective sgRNA was delivered by lentiviral vectors. Details on cloning of the lentiviral vectors are found in the Supplementary Materials.

For lentiviral vector production, 5 × 10^6^ HEK 293T cells were seeded on 10 cm plates in DMEM (Biochrom, Berlin, Germany) supplemented with 10% FBS (PanBiotech, Aidenach, Germany), 100 U/mL penicillin (PanBiotech, Aidenach, Germany), 100 µg/mL streptomycin (PanBiotech, Aidenach, Germany), and 1 mM sodium pyruvate (PanBiotech, Aidenach, Germany). Lentiviral vector particles were produced by transfection of 10 µg vector plasmid, 12 µg pcDNA.GP.4xCTE (encoding lentiviral Gag/Pol proteins) [23], 5 µg pRSV.Rev (kindly provided by T. Hope, Northwestern University Chicago, IL, USA), and 2 µg pMD.G (VSVg) [24] using the calcium-phosphate method as described elsewhere [25]. The lentiviral vector particles were concentrated via ultracentrifugation, resuspended in StemSpan (Stem Cell Technologies, Vancouver, BC, Canada) and stored at −80 °C.

The lentiviral vectors were titrated on lineage-depleted murine HSPCs to achieve an equal transduction rate between knockout and competitor cells for the subsequent competitive bone marrow transplantation.

### 2.3. Bone Marrow Transplantation

Murine bone marrow cells were isolated by flushing femurs, tibiae, and pelvis with MACS buffer (PBS supplemented with 0.5% BSA (PanBiotech, Aidenach, Germany) and 1 mM EDTA (Thermo Fisher Scientific, Waltham, MA, USA)). The bone marrow was passed through a 70 µm filter (Thermo Fisher Scientific, San Diego, CA, USA) to obtain single cells and incubated for 10 min in red blood cell lysis buffer to remove erythrocytes. Lineage depletion was performed using the MojoSort Mouse Hematopoietic Progenitor Cell Isolation Kit (BioLegend, San Diego, CA, USA) according to the manufacturer’s instructions. Lineage-negative bone marrow cells were cultured in HSPC medium (StemSpan supplemented with 100 U/mL penicillin, 100 µg/mL streptomycin, 2 mM L-glutamine (Biochrom, Berlin, Germany), 20 ng/mL mTPO (Peprotech, Hamburg, Germany), 20 ng/mL mIGF2 (Peprotech, Hamburg, Germany), 10 ng/mL mSCF (Peprotech, Hamburg, Germany), 10 ng/mL hFGF1 (Peprotech, Hamburg, Germany), 20 µg/mL Meropenem (Hexal, Holzkirchen, Germany), and 25 U/mL heparin (Ratiopharm, Ulm, Germany)) in a density of 1.5 × 10^6^ cells/mL.

For competitive bone marrow transplantations with knockout cells, HSPCs derived from Cas9 mice were transduced one day after isolation with lentiviral particles expressing a sgRNA and a fluorescent reporter (pRRL.PPT.hU6.sgRNA.EFS.dTomato.pre or pRRL.PPT.hU6.sgRNA.EFS.eBFP2.pre) in the presence of 4 µg/mL protamine sulfate (Sigma Aldrich, Steinheim, Germany) to enhance gene transfer. The cells were transduced overnight on two consecutive days with an MOI of 30. On the day of transplantation, equal cell numbers of lineage-negative bone marrows cells transduced with the targeting sgRNA and a dTomato fluorescence protein and competitor cells transduced with the nontargeting sgRNA and an eBFP2 fluorescence reporter were mixed (about 5–8 × 10^5^ cells per animal in total) and injected intravenously in 100 µL PBS per mouse. To determine the actual transduction rate, an aliquot of the cell mix was cultured for three days and analyzed by flow cytometry. CD45.1^+^ recipient mice were irradiated 24 h before transplantation with a single dose of 9 Gy or a fractionated dose of 2 × 4.5 Gy (5-h break in between the irradiations) to study effect of the irradiation regimen on the engraftment. Untransplanted, age-matched Cas9 mice were used as a control group in the final analysis.

For experiments with IL1B-challenged donor cells, CD45.2^+^ donor mice were injected five times over a period of 10 days intraperitoneally with 250 ng mIL1B (Peprotech, Hamburg, Germany) in 100 µL PBS or PBS alone as control before isolation of HSPCs as described above. To study the effect of p38 inhibition, donor cells were cultured for 2.5 days in HSPC medium supplemented with 10 µM SB203580 (p38-inhibitor (short: p38i)) (Axon Medchem, Groningen, The Netherlands) or 0.1% DMSO (Merck, Darmstadt, Germany) as vehicle control. Equal cell numbers of treated CD45.2^+^ donor cells (PBS + vehicle, IL1B + vehicle, PBS + p38i, IL1B + p38i) and untreated CD45.1^+^ competitors, which were cultured for 2.5 days in HSPC medium without any additional supplement, were mixed and transplanted into irradiated (9 Gy or 2 × 4.5 Gy) CD45.1^+^ recipients.

For the transplantation of X-CGD cells, HSPCs derived from the CD45.2^+^ X-CGD mouse model [26] (bone marrow samples were kindly provided by Katrin Schroeder, University of Frankfurt, Germany) were cultivated for 2.5 days with 10 µM SB203580 (p38i) or 0.1% DMSO as vehicle control. After cultivation, equal numbers of treated X-CGD HSPCs were mixed with untreated CD45.1^+^ HSPCs as competitor cells and transplanted into irradiated (9 Gy or 2 × 4.5 Gy) CD45.1^+^ recipients as described above.

The final analysis of transplanted animals was performed 18 weeks after transplantation.

### 2.4. Next Generation Sequencing

The enrichment of sgRNAs after transplantation of HSPCs, which were transduced with our targeted lentiviral sgRNA library, was analyzed in bone marrow samples 18 weeks after transplantation via Ion Torrent next-generation sequencing. Each sgRNA was identified via its unique 20 bp target sequence, which was amplified from genomic DNA via PCR. Genomic DNA of 5 × 10^6^ cells was isolated using the QIAamp DNA Blood Mini Kit (Qiagen, Hilden, Germany) according to the manufacturer’s instruction. The MyFi PCR mix (BioLine, Luckenwalde, Germany) was used to amplify the sgRNA target sequence using the primers sgRNA_NGS_fw (GGTGGGTTTTCCAGTCACACTGGACTATCATATGCTTACCG) and sgRNA_NGS_rev (TTCGTTGGGAGTGAATTAGCCACCGGAGCCACTCGAGGAATTC). The *Egr1* locus was amplified using the primers Egr1_NGS_fw (GGTGGGTTTTCCAGTCACACCCACCATGGACAACTACCCC) and Egr1_NGS_rev (TTCGTTGGGAGTGAATTAGCCGGCTCCCCTTGAGGATTGAA). In a second PCR, index sequences were added to each PCR product using 1 µL diluted PCR product (1:300), the pRGB_common primer and a distinct index primer (ITRGB_009-021) [27]. All samples were pooled for Ion Torrent sequencing. The sequencing results were assigned to each sample via their index sequence using a custom Perl 5 script (https://www.perl.org). In a second step, the presence of the respective target sequence for the sgRNA Lnk (GCGCGGTAGTCGTGACCCGG), p18 (GACGCCAACTCGTTCCCCCA), p38 (TGGATGTGTTCACACCCGCA), Egr1 (GGTGCTGCCGGAACCCCAGA), Ikkb (GGCCACAGCAGTTCTCGAAC), p190 (TATCATGATACAAGCGTACG), M40 (CTACAACCAGTGCAAATTCG), Gli1 (TCGACCTGCAAACCGTAATC), Cbl (CCCGTGGAAGAGCTTTCGAC), GzmB (TGGGGGCTTCCTTATTCGAG), and NT (GGCGTAGGTGTACGACCGCT) was quantified.

### 2.5. Flow Cytometry

Prior to antibody staining, all cells were blocked with a murine Fc receptor blocking reagent (BioLegend, San Diego, CA, USA) for 5–10 min at 4 °C. Surface staining was subsequently performed for 30 min at 4 °C. Live/dead staining was included to exclude dead cells using Zombie Aqua (BioLegend, San Diego, CA, USA), DAPI (Sigma Aldrich, Steinheim, Germany) or 7-AAD (BioLegend, San Diego, CA, USA). Apoptotic cells were detected by staining cells with Annexin V-APC (BioLegend, San Diego, CA, USA) in 1x Annexin V Binding Buffer (BioLegend, San Diego, CA, USA) for 15 min. The following antibodies were used: CD117 (c-Kit)-PE-Cy7 (2B8, Thermo Fisher Scientific, Waltham, MA, USA), CD117 (c-Kit)-PE (2B8, eBioscience/Thermo Fisher Scientific, Waltham, MA, USA), CD11b-APC (M1/70, eBioscience/Thermo Fisher Scientific, Waltham, MA, USA), CD150-APC (TC15-12F12.2, BioLegend, San Diego, CA, USA), CD3e-PE-Cy7 (145-2C11, eBioscience/Thermo Fisher Scientific, Waltham, MA, USA), CD45.1-APC eFluor780 (A20, eBioscience/Thermo Fisher Scientific, Waltham, MA, USA), CD45.2-Alexa Fluor 700 (104, BioLegend, San Diego, CA, USA), CD45R (B220)-PerCP-Cy5.5 (RA3.6B2, eBioscience/Thermo Fisher Scientific, Waltham, MA, USA), CD48-PE-Cy7 (HM48-1, eBioscience/Thermo Fisher Scientific, Waltham, MA, USA), CXCR4-APC (L276F12, BioLegend, San Diego, CA, USA), Lineage mix-Biotin (Caltag Medsystems, Buckingham, UK), Sca-1-APC (D7, BioLegend, San Diego, CA, USA), Sca-1-Alexa Fluor 700 (D7, eBioscience/Thermo Fisher Scientific, Waltham, MA, USA), and Streptavidin-PerCP-Cy5.5 (eBioscience/Thermo Fisher Scientific, Waltham, MA, USA). Intracellular phospho-p38 staining was performed after fixation and permeabilization (BioLegend, San Diego, CA, USA) with phospho-p38 (Cell Signaling Technologies, Danvers, MA, USA) as primary antibody and mouse-anti-rabbit Alexa Fluor 488 (Abcam, Cambridge, UK) as secondary antibody. Data was acquired at the LSR II (BD, Franklin Lakes, NJ, USA) or CytoFlex S (Beckman Coulter, Brea, CA, USA) and analyzed using the software FlowJo 10 (BD, Franklin Lakes, NJ, USA).

### 2.6. Western Blot

Bone marrow cells from *p38* knockout or control animals were lysed in 100 µL complete lysis buffer (50 mM Tris-HCl, 100 mM NaCl, 100 mM NaF, 1% Triton X-100, 100 mM dithiothreitol, 100 mM Na_3_VO_4_ and 1× Protease Inhibitor (complete Mini, Roche, Basel, Switzerland)) for 20 min on ice. The protein concentration in the supernatant was determined using the Bradford assay (Protein Assay Dye Reagent Concentrate, Bio-Rad, Hercules, CA, USA). Protein samples were separated on a 12% polyacrylamide gel and blotted onto a nitrocellulose membrane (Whatman Protran BA 85 Nitrocellulose). The membrane was blocked in 5% milk in TBST for 1 h at room temperature. Primary antibody staining (p38, clone: E229, Abcam, Cambridge, UK; Cbl, clone: A-9, Santa Cruz, Dallas, TX, USA and Lnk, clone: A-12, Santa Cruz, Dallas, TX, USA) was performed overnight at 4 °C. A horseradish peroxidase-conjugated secondary antibody (goat-anti-rabbit-HRP or goat-anti-mouse-HRP, Santa Cruz, Dallas, TX, USA) was applied for 1 h as a secondary antibody. GAPDH-HRP (clone: GT239, Genetex, Irvine, CA, USA) was used as loading control. Signals were detected using the SuperSignal West Pico Chemiluminescent Substrate (Thermo Fisher Scientific, Waltham, MA, USA) and the FusionX detection system (Peqlab, Erlangen, Germany).

### 2.7. Colony-Forming Unit (CFU) Assay

For the CFU assay, 1–2 × 10^4^ bone marrow cells were seeded in MethoCult^TM^ GF M3434 methylcellulose medium (StemCell Technologies, Vancouver, BC, Canada) according to the manufacturer’s instructions. Each sample was prepared in triplicate and seeded in 35 mm culture dishes. The dishes were placed in a humidified box and incubated at 37 °C and 5% CO_2_. After 14 days, the numbers of burst-forming unit-erythroid (BFU-E), colony-forming unit-granulocyte, monocyte (CFU-GM) and colony-forming unit-granulocyte, erythrocyte, monocyte, megakaryocyte (CFU-GEMM) colonies were counted using an inverted microscope Olympus SZ61 (Olympus, Tokyo, Japan).

### 2.8. Quantitative RT-PCR

RNA was isolated using the RNeasy Mini Kit (Qiagen) according to the manufacturer’s instruction. For each sample, 0.5 µg RNA was reverse transcribed into cDNA using the QuantiTect Reverse Transcription Kit (Qiagen, Hilden, Germany) following the manufacturer’s manual. The quantitative RT-PCR was performed in triplicates using the Step One Plus Real-Time PCR System (Thermo Fisher Scientific, Waltham, MA, USA) and the QuantiTect SYBR Green PCR Kit (Qiagen, Hilden, Germany). Expression of *p16* (p16_fw: GTGTGCATGACGTGCGGG; p16_rev: GCAGTTCGAATCTGCACCGTAG) and *p19* (p19_fw: GCCGCACCGGAATCCT; p19_rev: TTGAGCAGAAGAGCTGCTACGT) was normalized to *β-Actin* (β-Actin_fw: CCTCCCTGGAGAAGAGCTA; β-Actin_rev: TCCATACCCAAGAAGGAAGG). The data were analyzed using the ΔΔCt method and were presented as cells treated with a p38 inhibitor relative to cells treated with DMSO as vehicle control.

### 2.9. Statistics

All bar graphs represent the mean ± SD. Statistical significance was determined using a two-sided *t*-test, a multiple *t*-test correcting for multiple comparisons using the Holm–Sidak method or a two-way ANOVA with a Sidak post-hoc test and the software GraphPad Prism 7 (GraphPad Prism Incorporation, San Diego, CA, USA). All significant differences are indicated by * *p* ≤ 0.05, ** *p* ≤ 0.01, *** *p* ≤ 0.001, or **** *p* ≤ 0.0001. Not significant values are not shown or indicated by the exact *p*-value.

## 3. Results

### 3.1. Targeted Competitive sgRNA Screen Identifies Candidate Genes That Increase the Repopulating Capacity of HSPCs

**Figure 1:**
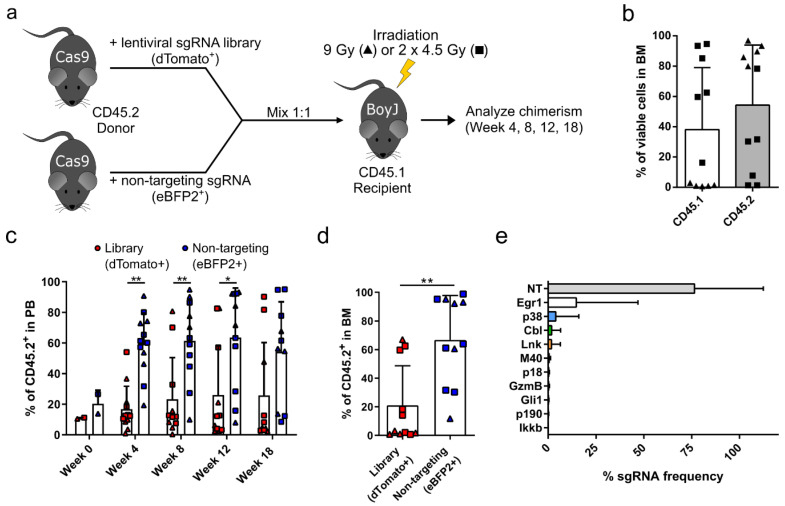
Targeted sgRNA screen identified potential candidate genes that mediate a competitive advantage during bone marrow transplantation. (**a**) Scheme of competitive bone marrow transplantation. Donor cells were derived from CD45.2^+^ Cas9 mice and were transduced with a lentiviral sgRNA library and a dTomato fluorescent reporter. CD45.2^+^ Cas9 competitor cells were transduced with a nontargeting sgRNA and eBFP2 fluorescent reporter. Donor cells were mixed 1:1 and transplanted into irradiated CD45.1^+^ recipient mice. Recipient mice were irradiated with either a single dose of 9 Gy (triangles) or a fractionated dose of 2 × 4.5 Gy (squares). Peripheral blood of the transplanted mice was analyzed on weeks 4, 8, and 12 after transplantation. Final analysis was performed 18 weeks after transplantation. (**b**) Donor cell chimerism was analyzed in the bone marrow 18 weeks after transplantation. (**c**) The presence of dTomato^+^ cells (red) carrying the sgRNA library and eBFP2^+^ competitor cells (blue) was monitored over time in peripheral blood. (**d**) The frequency of dTomato^+^ cells carrying the sgRNA library and eBFP2^+^ competitor cells was analyzed in the bone marrow on week 18 after transplantation. (**e**) The sgRNA frequency of each candidate gene in the library was assessed via next-generation sequencing in bone marrow samples of recipient mice 18 weeks after transplantation. NT = nontargeting. Statistics: *n* = 11, mean ± SD, two independent experiments, *t*-test (**b**,**d**), two-way ANOVA comparing row means only (**c**), * *p* < 0.05, ** *p* < 0.01.

To identify potential candidate genes whose inhibition can increase the repopulating capacity of HSPCs, we performed a small targeted sgRNA library screen using the CRISPR-Cas9 system. The candidates were chosen from the literature based on the following criteria: (1) the knockout increased the repopulating capacity of HSCs, (2) the knockout enhanced HSC frequency and self-renewal, and (3) the knockout did not cause tumorigenesis in serial transplantations. Based on these criteria, we chose 10 candidate genes, namely *Lnk* [21,28,29], *p18Ink4c* (*p18*) [30,31], *Mapk14* (*p38*) [32], *Egr1* [33], *Ikkb* [34], *p190-B* (*p190*) [35], *Merit40* (*M40*) [36,37,38], *Gli1* [39], *c-Cbl* (*Cbl*) [20], and *GzmB* [40]. Prior to the screen, each sgRNA of the library was independently tested in a fluorescent reporter assay for on-target cleavage activity (Figure S1a–c). The 10 sgRNAs of the library had an average on-target cleavage activity of 64%, and ranged between 46% for the sgRNA targeting *Ikkb* and 75% targeting *GzmB* (Figure S1d). After validation of each sgRNA separately, we generated the lentiviral sgRNA library by cotransfection of 11 different vector plasmids during virus production that carried sgRNAs targeting *Lnk*, *p18*, *p38*, *Egr1*, *Ikkb*, *p190*, *M40*, *Gli1*, *Cbl*, *GzmB*, and a nontargeting sgRNA (NT), which was added as an internal control. In contrast to the all-in-one CRISPR-Cas9 vectors used in the on-target reporter assay, the sgRNA only vectors achieved higher lentiviral titers and thus better transduction rates in primary murine HSPCs used for in vivo experiments. Thus, to induce a knockout, CD45.2^+^ HSPCs were derived from the Cas9 mouse model, which constitutively express Cas9. Cells were transduced on two consecutive days with the lentiviral sgRNA library, which is labeled with a dTomato fluorescent protein to track transduced cells. On day 3 after isolation, the cells were mixed in a 1:1 ratio with competitor cells (Cas9 HSPCs transduced with a nontargeting sgRNA and an eBFP2 fluorescent marker) and transplanted into CD45.1^+^ recipients (Figure 1a). Recipient mice were either irradiated with a single dose of 9 Gy or with a fractionated dose of 2 × 4.5 Gy to analyze the effect of irradiation on the engraftment of HSPCs in the bone marrow. On week 18 after transplantation, the donor chimerism was analyzed in the bone marrow of transplanted mice using the markers CD45.2 and CD45.1 to identify donor and recipient cells, respectively (Figure 1b). We observed a high donor cell engraftment for the 9 Gy irradiation cohort (triangles) and a mixed chimerism for the 2 × 4.5 Gy cohort (squares), with an overall donor content of 54.2% ± 39.6%. The presence of dTomato^+^ cells carrying the sgRNA library was monitored over time in the peripheral blood of transplanted animals. Starting as early as four weeks post-transplantation, three mice showed an increased presence of dTomato^+^ cells compared to eBFP2^+^ competitors (Figure 1c). However, in most animals, the eBFP2^+^ competitor cells dominated the hematopoiesis, indicating that most sgRNAs had no or a negative effect on the repopulating capacity of transplanted HSPCs. In the bone marrow of transplanted animals, the same three mice also showed an increased presence of dTomato^+^ cells, which reached about 60% of total CD45.2^+^ cells (Figure 1d). Total bone marrow samples of transplanted mice were subjected to next-generation sequencing to identify the frequency of each sgRNA in the bone marrow. The sequencing results revealed that the nontargeting sgRNA was most abundant (76.5% ± 36%), which is in line with the high frequency of eBFP2^+^ cells detected in the bone marrow of transplanted mice (Figure 1e). The next most frequent sgRNAs identified in the bone marrow of transplanted mice were targeting *Egr1* (14.7% ± 32.2%), *p38* (4.0% ± 12.1%), *Cbl* (1.8% ± 4.7%), and *Lnk* (1.7% ± 4.7%). These four candidates were validated in separate knockout experiments to study their effect on HSPC engraftment.

### 3.2. p38 Knockout HSPCs Significantly Outcompete Their Competitor Cells in a Bone Marrow Transplantation Assay

**Figure 2:**
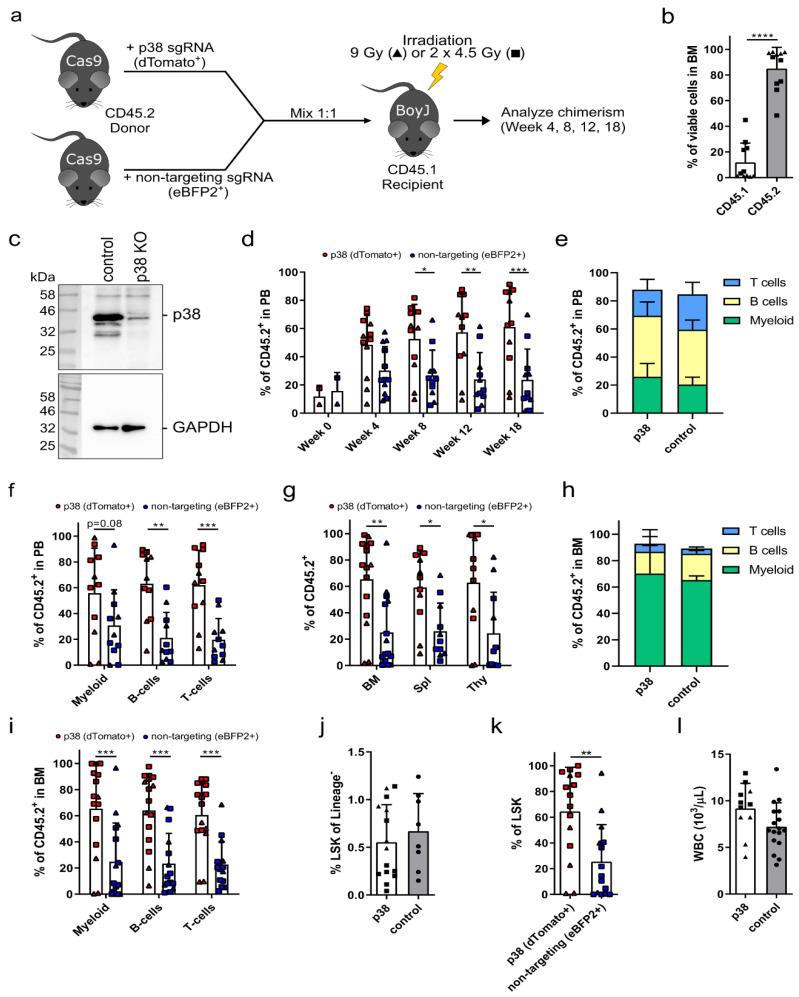
CRISPR-Cas9-mediated knockout of *p38* increases the repopulating capacity of hematopoietic stem and progenitor cells (HSPCs). (**a**) Schemata of the competitive bone marrow transplantation. Using lentiviral vectors, *p38* was knocked out in CD45.2^+^ HSPCs from Cas9 mice (labeled with a dTomato fluorescent reporter) and transplanted into irradiated CD45.1 mice. As competitors, HSPCs were transduced with a nontargeting sgRNA and an eBFP2 fluorescent reporter. Mice were irradiated with either a single dose of 9 Gy (triangles) or a fractionated dose of 2 × 4.5 Gy (squares). Peripheral blood was analyzed 4, 8, and 12 weeks after transplantation. On week 18, the animals were euthanized for final analysis. (**b**) The donor cell chimerism was analyzed as CD45.1^+^ and CD45.2^+^ cells in the bone marrow 18 weeks after transplantation. (**c**) The knockout of *p38* was verified by Western blot analysis of a representative bone marrow sample. GAPDH was used as loading control. KO = knockout. (**d**) The frequency of *p38* knockout HSPCs (red) and competitor cells (blue) was monitored over time in the peripheral blood of transplanted animals. (**e**) The lineage distribution for myeloid (CD11b^+^), B (B220^+^), and T (CD3^+^) cells was assessed in the peripheral blood of transplanted animals 18 weeks after transplantation in comparison to age-matched control animals. (**f**) The frequency of dTomato^+^ *p38* knockout cells (red) and eBFP2^+^ competitor cells (blue) was measured in myeloid, B, and T cells in the peripheral blood on week 18 after transplantation. (**g**) Bone marrow (BM), spleen (Spl), and thymus (Thy) were analyzed for the presence of *p38* knockout cells (red) and competitor cells (blue) 18 weeks after transplantation. (**h**) The lineage distribution in the bone marrow was analyzed 18 weeks after transplantation by measuring CD11b^+^ myeloid cells, B220^+^ B cells, and CD3^+^ T cells. (**i**) The presence of dTomato^+^ *p38* knockout cells and eBFP2^+^ competitor cells were assessed in myeloid, B, and T cells in the bone marrow of transplanted animals on week 18 post-transplantation. (**j**) The frequency of Sca1^+^ c-Kit^+^ Lineage^-^ (LSK) cells was measured in the bone marrow 18 weeks after transplantation. Circles refer to untransplanted age-matched control animals. (**k**) On week 18 post-transplantation, the frequency of dTomato^+^ *p38* knockout cells (red) and eBFP2^+^ competitor cells (blue) were analyzed in LSK cells in the bone marrow of transplanted mice. (**l**) The white blood cell count (WBC) was quantified in the peripheral blood in mice transplanted with *p38* knockout cells on week 18 after transplantation and compared to age-matched, untransplanted control animals (circles). Statistics: *n* = 11, mean ± SD, two independent experiments, *t*-test (**b**,**j**–**l**), two-way ANOVA comparing row means (**d**,**f**,**g**,**i**) or column means only (**e**,**h**), * *p* < 0.05, ** *p* < 0.01, *** *p* < 0.001, **** *p* < 0.0001.

Each of the four identified candidate genes *Egr1*, *p38*, *Cbl*, and *Lnk* was separately validated in a competitive transplantation assay as described above for the sgRNA screen. Briefly, dTomato^+^ knockout HSPCs were mixed in a 1:1 ratio with eBFP2^+^ competitor cells and transplanted into CD45.1^+^ recipients, which were irradiated with either 9 Gy or 2 × 4.5 Gy (Figure 2a, Supplementary Figures S2a, S3a, and S4a). Upon transplantation of *Egr1* knockout HSPCs, the average total CD45.2 engraftment, especially in the 2 × 4.5 Gy irradiation cohort, was higher (up to 81% ± 23.2% CD45.2^+^ cells (shown in Supplementary Figure S2b)) compared to the library screen (54.2% ± 39.6% CD45.2^+^ cells (data depicted in Figure 1b)). Deep sequencing confirmed indel formation at the Cas9 cleavage site in the *Egr1* locus in mice carrying the Egr1 sgRNA (Supplementary Figure S2c). However, transplanted animals did not show an increased presence of dTomato^+^ *Egr1* knockout cells in the peripheral blood over time (Supplementary Figure S2d). Comparison of lineage distributions between *Egr1* knockout cell transplanted animals and untransplanted age-matched control animals showed that the myeloid compartment was increased in mice that received *Egr1* knockout HSPCs, while B and T cells were similar in the peripheral blood of the two groups (Supplementary Figure S2e). Similarly, as observed for total CD45.2^+^ cells, no significant differences between *Egr1* knockout and competitor cells were observed in these different lineages 18 weeks after transplantation (Supplementary Figure S2f). This observation was also confirmed in other hematopoietic organs, such as spleen and thymus (Supplementary Figure S2g) and in greater detail in the bone marrow (Supplementary Figure S2h–j). Moreover, the frequency of Lineage^-^ Sca1^+^ c-Kit^+^ (LSK) cells, a marker combination for HSPCs, was significantly reduced in these animals (0.13% ± 0.08% Sca1^+^/c-Kit^+^ in lineage^−^ cells) compared to untransplanted age-matched controls (0.67% ± 0.39% Sca1^+^/c-Kit^+^ in lineage^−^ cells), indicating a rather negative effect on HSC self-renewal (Supplementary Figure S2j). Despite a reduced LSK frequency, we observed a slight, but not significant, increase of dTomato^+^ *Egr1* knockout cells in LSK cells (56.6% ± 40.7% dTomato^+^ cells) compared to eBFP2^+^ competitors (26.3% ± 37.7% eBFP2^+^ cells) (Supplementary Figure S2k) and a normal WBC count (Supplementary Figure S2l). Due to the reduced LSK frequency, the lack of dTomato^+^ cells in more mature cells and literature indicating a lack of a selective advantage during serial transplantation of *Egr1* knockout cells [33], we did not further follow up on *Egr1* as a candidate.

Thus, we next focused on *p38* as the second-best hit from our pooled sgRNA screen. Upon competitive transplantation of *p38* knockout HSPCs into irradiated recipients (Figure 2a), we observed an overall CD45.2 donor cell engraftment of up to 85.1% ± 16.4% CD45.2^+^ cells (Figure 2b). In general, all animals irradiated with a single dose of 9 Gy achieved a donor cell engraftment of over 90%. In contrast, engraftment after a fractionated irradiation was highly variable, most likely due to insufficient clearance of the bone marrow niche. Similarly to the *Egr1* group (Supplementary Figure S2b), *p38* knockout cell transplanted animals that received a fractionated irradiation regimen had a higher donor cell engraftment (Figure 2b) compared to the cohort in the library screen (Figure 1b), indicating that the knockout had a positive effect on the overall donor chimerism. The loss of p38 was confirmed in whole bone marrow samples 18 weeks after transplantation by Western blot, which revealed almost complete absence of p38 protein (Figure 2c). The residual p38 protein expression was most likely derived from the competitors and endogenous CD45.1^+^ cells. Over time in the peripheral blood, we observed that dTomato^+^ *p38* knockout cells outcompeted the eBFP2^+^ competitor cells (Figure 2d). Starting from week 8 onward, the frequency of dTomato^+^ *p38* knockout cells was significantly higher than the competitors and reached 61.1% ± 29% dTomato^+^ cells in week 18 compared to 23.7% ± 21.8% eBFP2^+^ cells. The lineage distribution of myeloid, B, and T cells was similar in the peripheral blood between *p38* knockout cell transplanted animals and untransplanted age-matched control animals (Figure 2e). With the exception of myeloid cells, the frequency of dTomato^+^ *p38* knockout cells was also significantly increased in B and T cell lineages in the peripheral blood compared to the competitors (Figure 2f). The dTomato^+^ *p38* knockout cells also dominated the hematopoiesis in the total CD45.2^+^ cells of the bone marrow, spleen, and thymus (Figure 2g). The bone marrow was analyzed in more detail and did not reveal any difference in the lineage distribution towards myeloid, B, or T cells in transplanted animals compared to the untransplanted controls (Figure 2h). In all three lineages, the frequency of dTomato^+^ *p38* knockout cells was significantly higher compared to competitors (Figure 2i). The frequency of HSPCs identified as LSK cells in the bone marrow was similar between *p38* knockout cell transplanted animals (0.55% ± 0.39% Sca1^+^/c-Kit^+^ in lineage^−^ cells) and untransplanted control animals (0.67% ± 0.39% Sca1^+^/c-Kit^+^ in lineage^−^ cells) (Figure 2j) despite an increased presence of dTomato^+^ *p38* knockout cells (64.5% ± 34.3% dTomato^+^ cells) compared to competitors (25.5% ± 28.7% eBFP2^+^ cells) (Figure 2k). Finally, the WBC count was normal in *p38* knockout cell transplanted animals (Figure 2l). In summary, we could validate knockout of *p38* as a potential target to improve the repopulating capacity of HSPCs during transplantation.

The other two candidate genes Cbl and Lnk were also validated in competitive bone marrow transplantation assays. Western blot results confirmed the loss of the respective proteins in bone marrow samples of knockout animals (Supplementary Figures S3c and S4c). Similarly to p38, the experiments confirmed that the knockout of each candidate had a positive effect on the repopulating capacity of HSPCs (Supplementary Figures S3 and S4). However, the transplantation of Cbl knockout HSPCs resulted in a significantly reduced frequency of LSK cells in the bone marrow of transplanted animals and an increased WBC count compared to untransplanted age-matched control animals (Supplementary Figure S3k,l). The performance of Lnk knockout HSPCs was superior to all other three candidates in terms of engraftment rates in the competitive transplantation. Competitor cells were almost completely outcompeted by Lnk knockout cells in various hematopoietic organs and subpopulations (Supplementary Figure S4d,f,g,i,k). Despite these positive results, the WBC count reached pre-leukemic levels in Lnk knockout cell transplanted animals (Supplementary Figure S4l). Thus, Cbl and Lnk might not be suitable candidates for a potential clinical translation. However, whether a transient inhibition of Cbl or Lnk using a small molecule inhibitor would be beneficial for the engraftment remains to be tested.

### 3.3. p38 Is a Druggable Target That Improves the Engraftment of HSPCs from IL1B-Challenged Donor Mice

**Figure 3:**
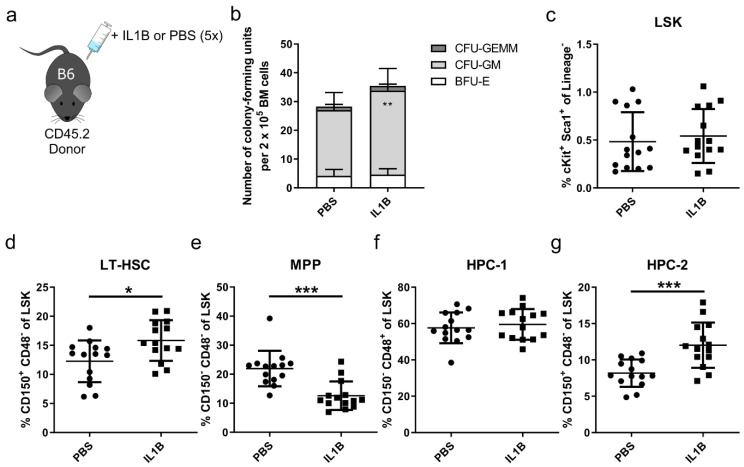
Challenging donor mice with IL1B leads to activation of LT-HSCs and mimics X-CGD disease pathology. (**a**) CD45.2^+^ donor mice were injected five times in a 2-day interval with IL1B or PBS as control. (**b**) Colony-forming assay was performed on bone marrow samples of IL1B- or PBS-treated donor mice. CFU = colony-forming unit, GEMM = granulocytic, erythroid, monocyte-macrophage, and megakaryocyte, GM = granulocyte/monocyte-macrophage, BFU = burst-forming unit, E = erythroid. Statistics: *n* = 4, mean ± SD, two-way ANOVA comparing row column means only, ** *p* < 0.01. (**c**–**g**) The frequency of (c) Sca1^+^ c-Kit^+^ Lineage^-^ cells (LSK), (d) CD150^+^ CD48^-^ long-term (LT-) HSCs, (**e**) CD150^-^ CD48^-^ multipotent progenitors (MPP), (**f**) CD150^-^ CD48^+^ hematopoietic progenitors type 1 (HPC-1), and (**g**) CD150^+^ CD48^+^ hematopoietic progenitors type 2 (HPC-2) was measured in the bone marrow of IL1B- and PBS-challenged mice. Statistics: *n* = 14, mean ± SD, four independent experiments, *t*-test, * *p* < 0.05, ** *p* < 0.01, *** *p* < 0.001.

For the translational perspective of interfering with p38, we aimed to enhance the repopulating capacity of HSPCs in a mouse model for X-CGD cells using a commercially available small molecule inhibitor (SB203580) instead of a genetic knockout. To mimic the HSC defect as observed in the bone marrow of X-CGD patients, we challenged CD45.2^+^ donor mice with IL1B prior to isolation of HSPCs as described by Weisser et al. (Figure 3a) [15]. To determine the frequency of HSPCs in the bone marrow, we performed colony-forming assays on isolated bone marrow cells of donor mice, which were treated five times with IL1B or PBS as control (Figure 3b). In comparison to PBS-treated control samples, IL1B-treated bone marrow cells revealed an increased frequency of CFU-GM colonies, which indicates an increased frequency of hematopoietic progenitor cells. Different HSPC subpopulations were further analyzed by staining LSK cells for CD150 and CD48 to detect the frequency of long-term hematopoietic stem cells (LT-HSCs; CD150^+^ CD48^-^), multipotent progenitors (MPPs; CD150^-^ CD48^-^), hematopoietic progenitor cells type 1 (HPC-1; CD150^-^ CD48^+^), and hematopoietic progenitor cells type 2 (HPC-2; CD150^+^ CD48^+^) (Figure 3c–g) [41]. The respective gating strategy is depicted in Supplementary Figure S5. Upon IL1B-treatment, we observed a significant increase of LT-HSCs and HPC-2 and a decrease of MPPs. These findings confirmed that pro-inflammatory cytokines, such as IL1B, can activate aberrant cell cycling in HSCs as shown by Weisser et al. [15], which presumably results in an expanded LT-HSC pool with compromised regenerative potential.

**Figure 4:**
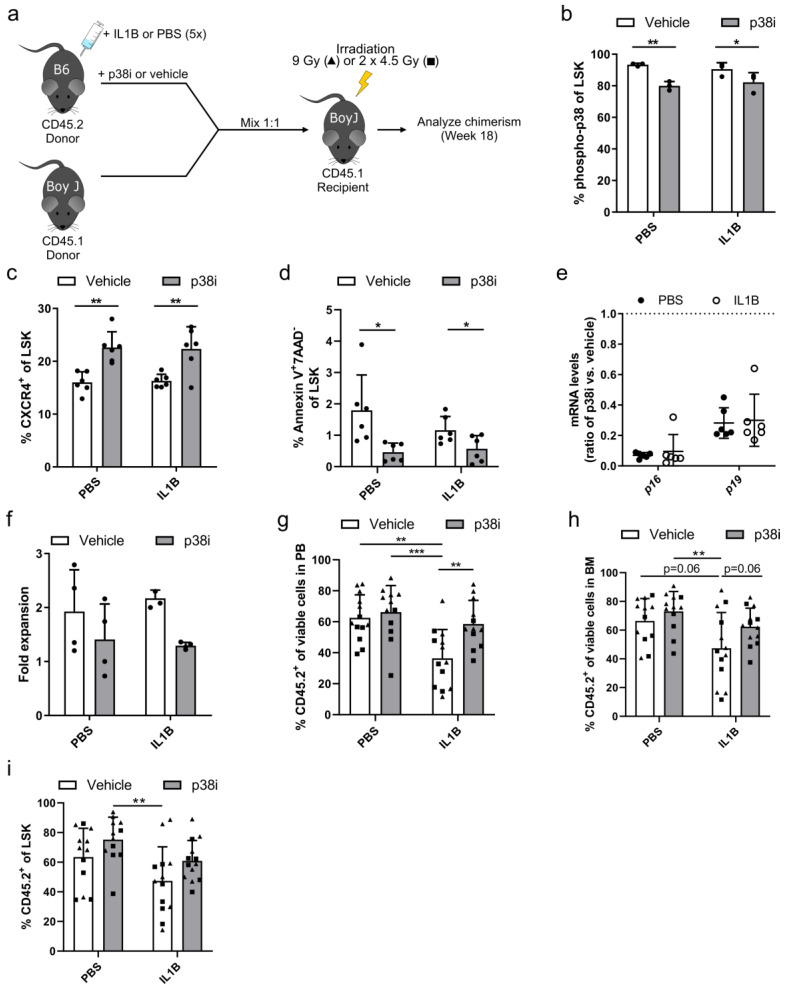
Inhibition of p38 MAPK improves the engraftment of IL1B-challenged HSPCs. (**a**) Schemata of competitive bone marrow transplantation after IL1B challenge. HSPCs derived from IL1B-challenged or PBS-control CD45.2^+^ donor mice were cultured for 2.5 days in the presence of a p38 small molecule inhibitor (p38i) or DMSO as vehicle control. Untreated CD45.1^+^ HSPCs were used as competitors. Donor cells were mixed in a 1:1 ratio and transplanted into irradiated CD45.1^+^ recipient mice. Recipients were irradiated either with a single dose of 9 Gy (triangles) or a fractionated dose of 2 × 4.5 Gy (squares). The animals were analyzed on week 18 after transplantation. (**b**) Intracellular staining was performed to detect phospho-p38 levels in IL1B-challenged or PBS-control LSK cells after cultivation of HSPCs with p38i or vehicle control. (**c**) Prior to transplantation, expression of the HSC homing factor CXCR4 was analyzed in LSK cells derived from IL1B-challenged or PBS-control donor mice after cultivation with p38i or DMSO as vehicle control. (**d**) Apoptotic cells were assessed in LSK cells treated with p38i or DMSO as vehicle control by staining for Annexin V^+^ and 7AAD^-^ cells. (**e**) Quantitative RT-PCR was performed to detect *p16* and *p19* mRNA levels in HSPCs treated with p38i in relation to vehicle-treated HSPCs. Expression levels were normalized to *β-Actin* levels. The dotted line at 1.0 indicates the level of normalization (no up- or downregulation). (**f**) The cell expansion during cultivation of IL1B-challenged or PBS-control HSPCs was quantified by comparing the cell count before and after treatment with p38i or DMSO as vehicle control. (**g**) The frequency of CD45.2^+^ donor cells was analyzed in the peripheral blood of animals 18 weeks after transplantation of p38i-treated or vehicle control HSPCs. (**h**) The CD45.2^+^ donor cell engraftment was measured in bone marrow samples of transplanted animals 18 weeks after transplantation of p38i-treated or vehicle control HSPCs. (**i**) The frequency of CD45.2^+^ donor cells was assessed in LSK cells on week 18 after transplantation in the bone marrow of mice transplanted with p38i-treated or DMSO-treated vehicle control HSPCs. Statistics: in vitro experiments (**b**–**f**): *n* = 3–6, mean ± SD, two-way ANOVA comparing row means only (**b,c,f**) or multiple *t*-test correcting for multiple comparisons using the Holm–Sidak method (**d**), in vivo experiments (**g**–**i**): *n* = 13, four independent experiments, mean ± SD, two-way ANOVA, * *p* < 0.05, ** *p* < 0.01, *** *p* < 0.001.

After induction of the HSC defect in donor mice via IL1B injection, which will become more obvious in subsequent transplantation experiments, IL1B-challenged and PBS-control HSPCs were cultured for 2.5 days in the presence of a p38 small molecule inhibitor (p38i) or DMSO as a vehicle control and transplanted in a competitive manner into irradiated CD45.1^+^ recipients using cultured, untreated CD45.1^+^ HSPCs as competitors (Figure 4a). Prior to transplantation, we confirmed the inhibition of p38 via intracellular staining of phospho-p38. In both IL1B-challenged and PBS-control HSPCs treated with p38i, we observed a reduced phosphorylation of p38 by 8.3% and 13.5% phospho-p38^+^ cells, respectively (Figure 4b). An important key factor in HSC engraftment is the expression of the chemokine receptor CXCR4, which mediates homing and retention of HSCs in the bone marrow niche [42]. Despite this low reduction in p38 phosphorylation, we observed a significant increase of CXCR4 expression of about 6% in LSK cells cultured with p38i in comparison to vehicle control samples (16.0% ± 2% and 16.3% ± 1.3% CXCR4^+^ cells to 22.6% ± 3% and 22.3% ± 4.2% CXCR4^+^ cells in PBS-controlled and IL1B-challenged samples, respectively) (Figure 4c). As p38 is also involved as a signal transducer in pathways that induce apoptosis, we measured the frequency of apoptotic cells in the HSPC cultures before transplantation. Compared to vehicle-treated cultures (1.8% ± 1.1% to 1.2% ± 0.4% apoptotic cells in PBS-control and IL1B-challenged, respectively) the p38i treatment resulted in reduced frequency of apoptotic cells (0.5% ± 0.3% to 0.6% ± 0.4% apoptotic cells in PBS-control and IL1B-challenged samples, respectively) as measured by Annexin V^+^/7-AAD^-^ cells in the LSK subpopulation (Figure 4d). Although the difference of 1% apoptotic cells might only have a minor effect on HSC engraftment, we observed a downregulation of *p16* and *p19* mRNA levels in p38i- compared to vehicle-treated samples, which are known inducers of apoptosis and senescence (Figure 4e) [32]. Finally, p38i-treated cells had a tendency for a lower proliferation during cultivation compared to vehicle control samples (Figure 4f). After studying the effect of p38i on cultured HSPCs *in vitro*, p38i-treated and vehicle-controlled HSPCs derived from either IL1B-challenged or PBS-control donor mice were transplanted into irradiated CD45.1^+^ recipients using cultured, untreated CD45.1^+^ cells as competitors. While the cultivation of HSPCs derived from PBS-control donors with p38i had no effect on the CD45.2^+^ donor cell engraftment, we observed a significant increase in the repopulating capacity of p38i-treated HSPCs from IL1B-challenged donor mice in the peripheral blood (Figure 4g). Due to the HSC defect in the vehicle-treated IL1B-challenged cohort, a donor cell chimerism of only 36.4% ± 18.5% CD45.2^+^ cells was achieved. The cultivation of HSPCs with p38i increased the engraftment 1.6-fold to 58.6% ± 15.2% CD45.2^+^ cells, which was comparable to vehicle-treated PBS-control HSPCs. A similar, but not significant, trend was observed in the bone marrow (*p* = 0.06) and LSK cells (*p* = 0.13) of transplanted animals (Figure 4h,i). In summary, we observed that p38 inhibition increased CXCR4 expression in HSPCs and reduced their apoptosis rate and proliferation, which might have a positive effect on the engraftment of p38i-treated cells, especially after IL1B exposure.

### 3.4. Inhibition of p38 Increases the Engraftment of HSPCs Derived from the X-CGD Mouse Model

**Figure 5:**
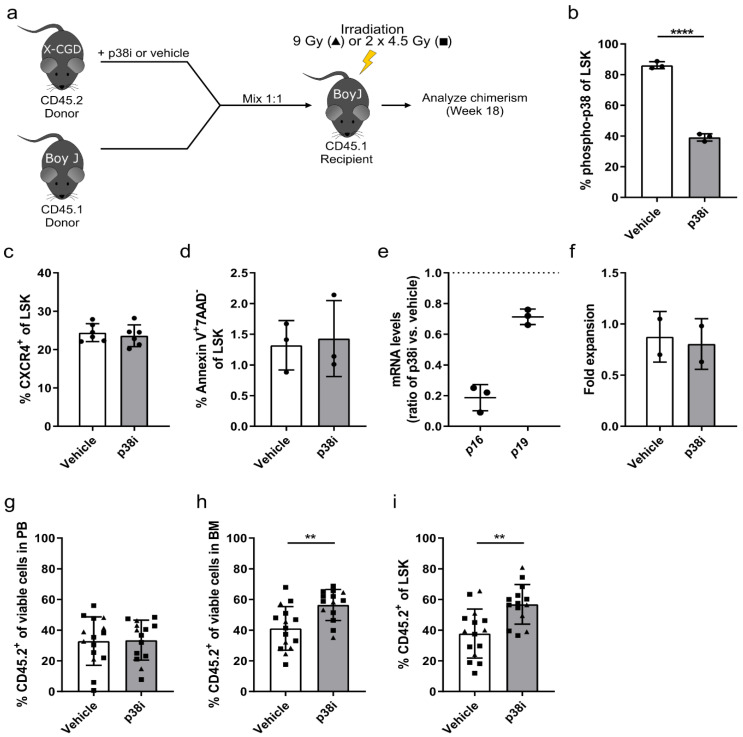
Inhibition of p38 MAPK improves the engraftment of HSPCs derived from the X-CGD mouse model. (**a**) Schemata of competitive bone marrow transplantation of X-CGD HSPCs. After isolation, HSPCs were cultured for 2.5 days in the presence of a p38 small molecule inhibitor (p38i) or DMSO as vehicle control. Untreated CD45.1^+^ HSPCs were used as competitors. Donor cells were mixed in a 1:1 ratio and transplanted into irradiated CD45.1^+^ recipient mice. Recipients were irradiated with either a single dose of 9 Gy (triangles) or a fractionated dose of 2 × 4.5 Gy (squares). The animals were analyzed on week 18 after transplantation. (**b**) Phospho-p38 levels were measured in X-CGD LSK cells after cultivation with p38i or vehicle control by intracellular staining. (**c**) Expression of the HSC homing factor CXCR4 was analyzed in X-CGD LSK cells after cultivation with p38i or DMSO as vehicle control prior to transplantation. (**d**) Apoptosis was measured in X-CGD LSK cells treated with p38i or DMSO as vehicle control by staining for Annexin V^+^ and 7AAD^-^ cells. (**e**) Quantitative RT-PCR was performed to detect *p16* and *p19* mRNA levels in X-CGD HSPCs treated with p38i in relation to vehicle-treated HSPCs. Expression levels were normalized to *β-Actin* levels. The dotted line at 1.0 indicates the level of normalization (no up- or downregulation). (**f**) The cell expansion of X-CGD HSPCs was quantified by comparing the cell count before and after treatment with p38i or DMSO as vehicle control. (**g**) The frequency of CD45.2^+^ X-CGD donor cells was analyzed in the peripheral blood of animals 18 weeks after transplantation of p38i-treated or vehicle control HSPCs. (**h**) The X-CGD CD45.2^+^ donor cell engraftment was assessed in bone marrow samples of transplanted animals 18 weeks after transplantation of p38i-treated or vehicle-control HSPCs. (**i**) On week 18 after transplantation, the frequency of CD45.2^+^ donor cells was measured in LSK cells in the bone marrow of mice transplanted with p38i-treated or DMSO-treated vehicle control X-CGD HSPCs. Statistics: in vitro experiments (**b**–**f**): *n* = 2–6, mean ± SD, in vivo experiments (**g**–**i**): *n* = 15, two independent experiments, mean ± SD, *t*-test, ** *p* < 0.01, **** *p* < 0.0001.

In contrast to the IL1B-induced acute inflammation of donor mice, HSCs from X-CGD are exposed to a chronically inflamed milieu during their entire lifetime presumably resulting in a more pronounced HSC defect [26]. We thus investigated the effect of p38 inhibition on X-CGD donor mice and cultured their HSPCs for 2.5 days in the presence of p38i or DMSO prior to competitive transplantation into irradiated CD45.1^+^ donor mice (Figure 5a). Cultured, untreated CD45.1^+^ HSPCs were used as competitor cells. Prior to transplantation, the level of p38 phosphorylation was assessed via intracellular staining. Upon inhibition of p38, we observed a two-fold reduction in phospho-p38 levels in p38i-treated (39.1% ± 2.4% phospho-p38^+^ cells) compared to vehicle-control X-CGD LSK cells (86.1% ± 2.3% phospho-p38^+^ cells) (Figure 5b). In contrast to LSK cells from IL1B-challenged mice, the inhibition of p38 had no effect on the expression of CXCR4 in the X-CGD LSK cells (Figure 5c). Moreover, the level of apoptotic cells did not differ between p38i-treated and vehicle-control X-CGD LSK cells (Figure 5d). However, we still observed a downregulation in the mRNA expression level of *p16* and *p19*, which are involved in the induction of senescence and apoptosis (Figure 5e). During cultivation of X-CGD HSPCs, we also observed no difference in the proliferative capacity of p38i-treated cells compared to vehicle controls (Figure 5f). These in vitro results were in strong contrast to the previous results of HSPCs from IL1B-challenged HSPCs. Despite the difference in observation, we transplanted the p38i-treated and vehicle-control cells into irradiated recipients and observed additionally no effect on the CD45.2 donor cell chimerism in the peripheral blood of transplanted animals 18 weeks post-transplantation (Figure 5g). However, the frequency of CD45.2^+^ donor cells was significantly increased in the bone marrow of mice that received p38i-treated cells (Figure 5h). Compared to vehicle-control samples, which achieved a donor chimerism of 41.2% ± 14.2% CD45.2^+^ cells, the engraftment of p38i-treated was increased by 1.4-fold to 56.5% ± 10.1% CD45.2^+^ donor cells. This finding was confirmed in the LSK subpopulation with a 1.5-fold increase from 37.8% ± 16% CD45.2^+^ cells in vehicle-control cells to 56.8% ± 12.9% CD45.2^+^ cells in p38i-treated cells (Figure 5i). The inhibition of p38 prior transplantation increased the engraftment capacity of X-CGD LSKs, indicating a positive effect on the repopulating capacity of HSCs. Together our data demonstrate the potential of p38i for restoring the repopulating capacity of HSPC grafts derived from a pro-inflammatory and chronically inflamed milieu.

## 4. Discussion

This study aimed to identify a target gene whose inhibition can improve the engraftment of HSPCs in the context of X-CGD gene therapy. In our competitive sgRNA screen, we identified and validated that loss of *p38*, *Cbl*, and *Lnk* expression can improve HSPC engraftment. As a genetic knockout is undesirable in a future clinical setting, we subsequently focused on p38 due to the availability of a small molecule inhibitor. We confirmed that inhibition of p38 during the ex vivo cultivation of HSPCs prior to transplantation can improve the repopulating capacity of HSPCs. The small molecule inhibitor had only a minor effect on healthy HSPCs in contrast to the CRISPR-Cas9 mediated *p38* knockout. However, upon p38 inhibition, we observed a significant increase in engraftment of HSPCs derived from a pro-inflammatory environment, such as observed in X-CGD mice. One main driver of HSC exhaustion in X-CGD patients is the pro-inflammatory cytokine IL1B, which activates cell cycling in HSPCs [15]. We mimicked this effect by challenging donor mice with IL1B prior to isolation of HSPCs. The inhibition of p38 in IL1B-challenged HSPCs increased expression of the homing factor CXCR4 and reduced apoptosis and proliferation. Despite these encouraging findings, we only observed a significantly increased engraftment of HSPCs in the peripheral blood of recipient mice but not in the bone marrow. Although there was a minor increase in donor cells in the bone marrow, the engraftment rates were highly variable in the untreated IL1B-challenged group, which might be caused by the short IL1B stimulation in donor mice, which lasted only 10 days. Some donor mice seem to have responded more sensitively to the IL1B treatment than others, which was the major source of the variability in engraftment rates. Moreover, based on the duration of IL1B exposure, we speculate that the short IL1B stimulation mimicked an acute rather than a chronic inflammation, which might have affected HSPCs to a different extent. In contrast, using HSPCs from X-CGD mice, we observed an increased donor cell engraftment in the bone marrow and stem cell compartment of recipient mice but not in the peripheral blood, indicating that the chronic inflammation or long-term exposure to pro-inflammatory cytokines in X-CGD mice might have a more pronounced effect on the HSCs.

A study by Weisser et al. showed that the engraftment of X-CGD HSPCs was improved in mice treated with Anakinra, an IL-1 receptor antagonist, confirming that the IL-1 signaling pathway is involved in the induction of the HSC defect [15]. In our studies, we identified p38 as another druggable downstream target of the IL-1 signaling pathway that restores the engraftment of X-CGD HSPCs. Besides pro-inflammatory cytokines, p38 is activated by several different stimuli, such as DNA damage and oxidative stress [43]. We hypothesize that inhibition of a downstream target such as p38 could be more beneficial than directly targeting the IL-1 receptor as p38 inhibition is expected to also result in blockade of other cytokine signaling pathways and oxidative stress during the ex vivo cultivation of HSCs under normoxic conditions. Moreover, direct inhibition of receptors may lead to broader effects, including inhibition of receptor activities that may be beneficial to HSC cultivation. Thus, blockade of downstream effectors known to control activities that are deleterious to HSC culture would provide a more specific and defined strategy to improve HSC culture and subsequent engraftment. Upon activation, the p38 MAPK pathway can lead to the induction of senescence and apoptosis in HSCs via upregulation of p16 and p19 [32], which consequently results in reduced HSC numbers and impaired long-term engraftment. Our data demonstrated that genetic or pharmacologic intervention of HSPCs shortly before transplantation was possible and can be exploited to enhance HSPC repopulation and engraftment capacity. Although the time window of ex vivo cultivation is very short in a gene therapy setting (usually less than 2–3 days) to avoid loss of stemness by extended cultivation, we confirmed that the manipulation of p38 during this time window is sufficient to improve the repopulating capacity of HSPCs in this specific context. Whether the treatment of patients with a p38 inhibitor before and/or after gene therapy is also beneficial for the long-term engraftment remains to be studied. Various p38 inhibitors are already tested in the clinics for the treatment of cancer or inflammatory conditions [44,45].

In our targeted screen, we also identified *Cbl* and *Lnk* as additional candidates to enhance the repopulating capacity of HSPCs. These genes, especially *Lnk*, were shown to play a role in various forms of leukemia and non-hematologic tumors [46,47]. Thus, we observed an increased number of white blood cells in mice transplanted with these knockout cells. It remains to be tested whether this also holds true for a transient inhibition during ex vivo cultivation, e.g., using a small molecule inhibitor or a transient shRNA approach. Moreover, it would be interesting to test whether the combination of two or three different inhibitors could potentiate the effect on the repopulating capacity or whether the genes function in a synergistic manner. In contrast to p38, Lnk and Cbl are known inhibitors of Tpo and c-Kit signaling in HSPCs, which play important roles in HSC self-renewal [21]. The inhibition of *Lnk* and *Cbl* enhances the sensitivity towards Tpo and stem cell factor, the c-Kit ligand, and results in an increased frequency of HSCs [20,36,38,48,49]. As the mechanism is different between *p38* and *Lnk*/*Cbl*, we hypothesize that a combination may be beneficial.

To further translate our findings into the clinic, the effect must be reproducible in human HSPCs derived from X-CGD patients. Previously, Zou et al. demonstrated that healthy human CD133^+^ HSPCs from umbilical cord blood treated with a p38 inhibitor revealed an increased expansion of HSCs and an increased engraftment after prolonged cultivation compared to DMSO-control samples in immunodeficient mice [50]. The ultimate goal for future gene therapies would be to transplant HSPCs with an engraftment advantage into a nonconditioned patient to avoid toxicity of the conditioning regimen. Currently, a complete myeloablative conditioning regimen is applied to X-CGD patients prior to infusion of gene-corrected cells to promote their long-term engraftment [9]. As a complete myeloablative conditioning regimen is quite toxic to the patient and can cause multiorgan damage, infertility, and secondary malignancies [51,52], long-term engraftment of corrected HSPCs without or after a reduced-intensity conditioning regimen is desirable for future gene therapies. Thus, to equip HSCs with a selective advantage is not only of interest for X-CGD gene therapy, but also for other pathologies that benefit from gene therapy or from bone marrow transplantation in general. Recently, HSCs with somatic mutations in *LNK* were identified in patients that responded with an improved myocardial regeneration after infarction and CD133^+^ cell/coronary artery bypass graft surgery [53]. The findings indicate that modulation of *LNK* function in HSCs might also be beneficial for non-hematologic disorders.

In summary, we hypothesize that the p38 MAPK pathway may play a role in the pro-inflammatory cytokine-mediated activation of HSCs, which results in lack of long-term engraftment after transplantation. The knockout or inhibition of p38 MAPK can partly counteract this HSC defect, which could be of therapeutic value for patients who are suffering from hyperinflammation and are undergoing gene therapy.

## Supplementary Materials

The following are available online at https://www.mdpi.com/2073-4409/9/10/2194/s1, Supplementary Methods, Figure S1: Evaluation of the lentiviral sgRNA library in a fluorescence reporter assay, Figure S2: CRISPR-Cas9-mediated knockout of *Egr1* does not increase the repopulating capacity of HSPCs, Figure S3: CRISPR-Cas9-mediated knockout of *Cbl* increases the engraftment of HSPCs in a competitive bone marrow transplantation, Figure S4: CRISPR-Cas9-mediated knockout of *Lnk* significantly increases the engraftment of HSPCs in a competitive bone marrow transplantation. Figure S5: Gating strategy for HSPC subpopulations.
