# Supplementary material for: Competitive sgRNA Screen Identifies p38 MAPK as a Druggable Target to Improve HSPC Engraftment
Source: Cells. 2020 Sep 29;9(10):2194. doi: 10.3390/cells9102194 (PMC7600420; doi:10.3390/cells9102194)
Supplement: Supplementary file 1 [file cells-09-02194-s001.pdf]

Supplementary Materials:

Supplementary Methods
Supplementary Figures

Figure S1: Evaluation of the lentiviral sgRNA library in a fluorescence reporter assay

Figure S2: CRISPR-Cas9-mediated knockout of *Egr1* does not increase the repopulating capacity of HSPCs

Figure S3: CRISPR-Cas9-mediated knockout of *Cbl* increases the engraftment of HSPCs in a competitive bone marrow transplantation

Figure S4: CRISPR-Cas9-mediated knockout of *Lnk* significantly increases the engraftment of HSPCs in a competitive bone marrow transplantation.

Figure S5: Gating strategy for HSPC subpopulations.

Supplementary Reference

Supplementary Methods

Cloning of lentiviral vectors expressing a sgRNA

The lentiviral vectors used to introduce a knockout contain a human U6 promoter that drives expression of the sgRNA. The sgRNA target sequences were designed using the CCTop online tool [1] and were cloned in front of the sgRNA scaffold using *BsmB*I recognition sites that are located within a filler sequence. During cloning of the target sequence, which consists of phosphorylated and annealed oligodeoxynucleotides, the filler sequence was replaced by the sgRNA target sequence. Additionally, the vectors contain a short EF1α promoter (EFS) that drives expression of a dTomato or eBFP2 fluorescent protein to track transduced cells. The vectors were named pRRL.PPT.hU6.filler-sgRNA.EFS.dTomato.pre and pRRL.PPT.hU6.filler-sgRNA.EFS.eBFP2.pre.

CRISPR-Cas9 fluorescence reporter assay

Prior to the application of an sgRNA in HSPCs, we tested all sgRNAs in fluorescence reporter assays. We cloned a lentiviral reporter construct for each target gene by cloning of the sgRNA target sequence in frame behind the start codon of a superfolder enhanced green fluorescent protein (sfGFP), which was followed by a puromycin resistance gene for selection. SC-1 cells were transduced with the reporter constructs using lentiviral particles. Transduced cells were enriched via the resistance gene using 3 μg/mL puromycin (Invivogen, San Diego, CA, USA). The reporter cell lines were subsequently transduced with an all-in-one CRISPR-Cas9 lentiviral vector, which co-expresses the respective sgRNA, spCas9 and dTomato, a red fluorescent protein used to visualize gene-edited cells. Upon delivery of CRISPR-Cas9 into the reporter cell line, the Cas9 cleaves the target sequence 5′ of sfGFP, which results in a loss of fluorescence due to frameshift mutations after induction of a DNA double strand break and non-homologous end-joining. The on-target cleavage activity was assessed as the frequency of sfGFP$^-$ cells in the dTomato$^+$ fraction.

Supplementary Figures

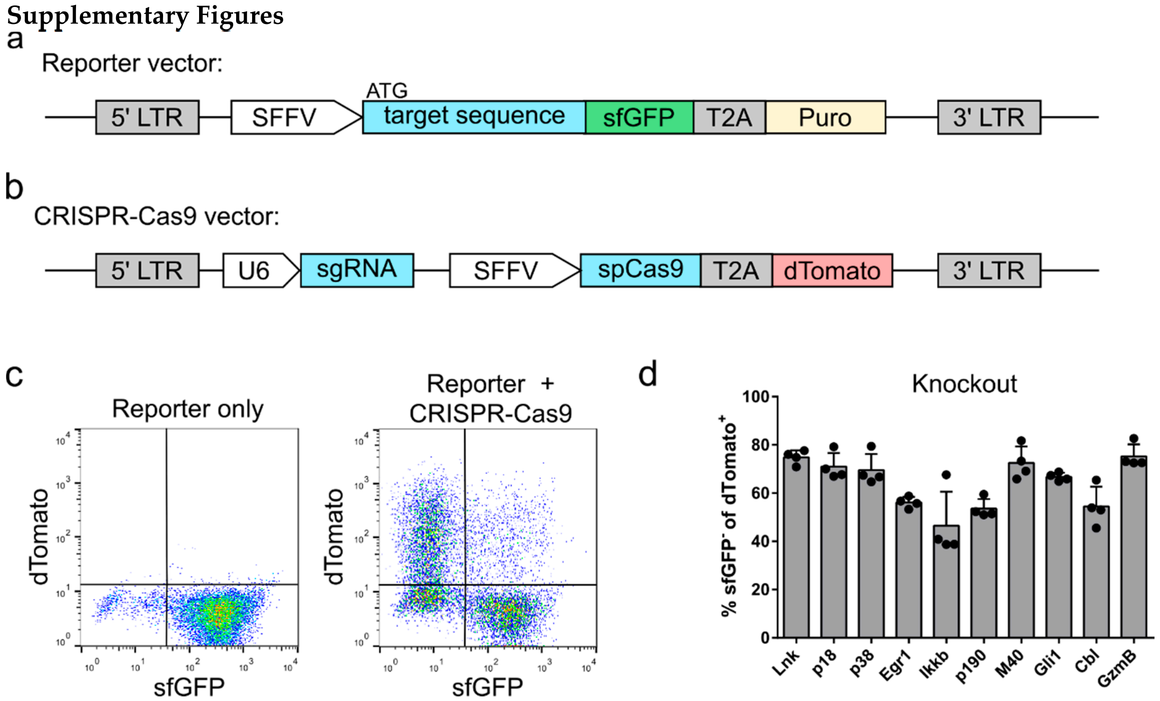

Supplement Figure S1. Evaluation of the lentiviral sgRNA library in a fluorescence reporter assay. (**a**) To test the sgRNAs for each target gene, a reporter cell line was generated by transduction of SC-1 cells with the depicted lentiviral vector. The vector contains a spleen focus-forming virus (SFFV) promoter-driven superfolder enhanced green fluorescent protein (sfGFP). The respective sgRNA target sequence was cloned behind the ATG in frame in front of the sfGFP followed by a puromycin resistance gene for selection. LTR: long-terminal repeat, T2A: Thosea asigna virus 2A peptide cleavage site. (**b**) All-in-one CRISPR-Cas9 vector used to test the sgRNA for each target gene. The sgRNA is driven by the human U6 promoter, which is followed by the SFFV promoter driving expression of the *Streptococcus pyogenes* Cas9 (spCas9) and a dTomato fluorescent protein. (**c**) Depicted are representative FACS dot plots of the reporter system. Left panel: Reporter cells prior to transduction with the CRISPR-Cas9 system. Right panel: Upon transduction of the reporter cells with the all-in-one CRISPR-Cas9 vector, the Cas9 cleaves the reporter gene, which results in the loss of sfGFP fluorescence. (**d**) The repair of the Cas9-induced DNA double strand break via non-homologous end-joining results in two out of three cases (~67%) in a frameshift and, thus, in loss of sfGFP fluorescence. The frequency of $sfGFP^-$ reporter cells (which is a surrogate marker for CRISPR-Cas9 activity) was measured in $dTomato^+$ cells (marker for cells transduced with the CRISPR-Cas9 vector) to analyze the sgRNA cleavage activity for each target gene.

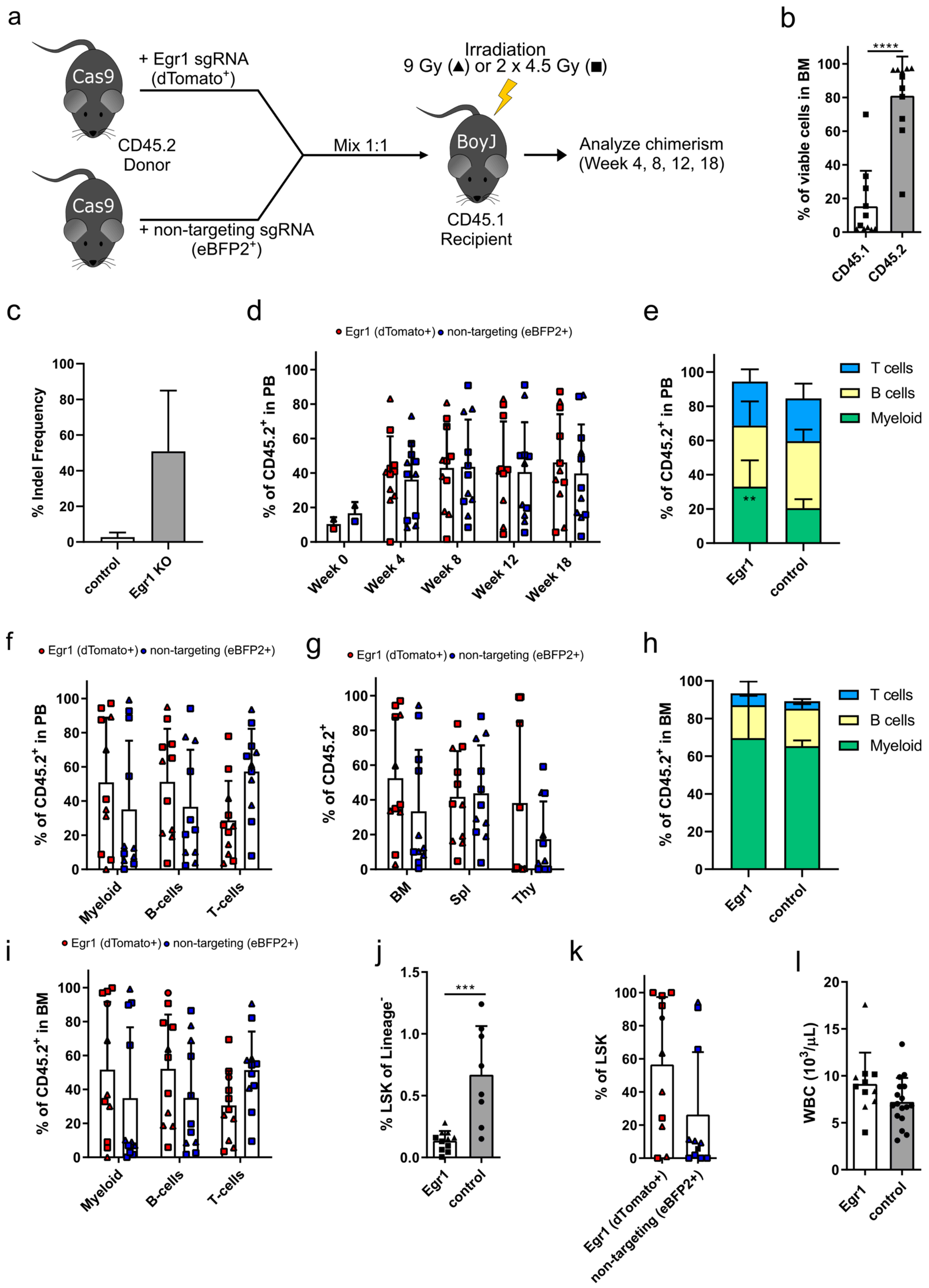

a
Cas9
+ Egr1 sgRNA (dTomato+)
CD45.2 Donor
Cas9
+ non-targeting sgRNA (eBFP2+)
Mix 1:1
Irradiation
9 Gy (▲) or 2 x 4.5 Gy (■)
BoyJ
CD45.1 Recipient
Analyze chimerism (Week 4, 8, 12, 18)
b
% of viable cells in BM
****
CD45.1
CD45.2
c
% Indel Frequency
control
Egr1 KO
d
Egr1 (dTomato+) non-targeting (eBFP2+)
% of CD45.2+ in PB
Week 0
Week 4
Week 8
Week 12
Week 18
e
% of CD45.2+ in PB
T cells
B cells
Myeloid
**
Egr1
control
f
Egr1 (dTomato+) non-targeting (eBFP2+)
% of CD45.2+ in PB
Myeloid
B-cells
T-cells
g
Egr1 (dTomato+) non-targeting (eBFP2+)
% of CD45.2+
BM
Spl
Thy
h
% of CD45.2+ in BM
T cells
B cells
Myeloid
Egr1
control
i
Egr1 (dTomato+) non-targeting (eBFP2+)
% of CD45.2+ in BM
Myeloid
B-cells
T-cells
j
% LSK of Lineage-
***
Egr1
control
k
% of LSK
Egr1 (dTomato+)
non-targeting (eBFP2+)
l
WBC (10^3/µL)
Egr1
control

Supplement Figure S2. CRISPR-Cas9-mediated knockout of *Egr1* does not increase the repopulating capacity of HSPCs. (**a**) Schemata of the competitive bone marrow transplantation performed to test the effect of *Egr1* knockout on HSPC engraftment. *Egr1* was knocked out in $CD45.2^+$ HSPCs from Cas9 mice using a lentiviral vector carrying the sgRNA and a dTomato fluorescent protein and transplanted into irradiated $CD45.1^+$ mice. As competitors, HSPCs were transduced with a lentiviral vector carrying a non-targeting sgRNA and an eBFP2 fluorescent protein. Mice were irradiated with either a single dose of 9 Gy (triangles) or a fractionated dose of 2 x 4.5 Gy (squares). Peripheral blood was analyzed 4, 8 and 12 weeks after transplantation. On week 18, the animals were euthanized for final analysis. (**b**) The donor cell chimerism was analyzed as the frequency of $CD45.1^+$ and $CD45.2^+$ cells in the bone marrow 18 weeks after transplantation. (**c**) Cleavage of the Cas9 target site was analyzed by deep sequencing in control mice and animals carrying the *Egr1* sgRNA (Egr1 KO). (**d**) The frequency of *Egr1* knockout HSPCs (red) and competitor cells (blue) was monitored over time in the peripheral blood of transplanted animals. (**e**) The lineage distribution for myeloid ($CD11b^+$), B ($B220^+$) and T ($CD3^+$) cells was assessed in the peripheral blood of transplanted animals 18 weeks after transplantation in comparison to age-matched control animals. (**f**) The frequency of $dTomato^+$ *Egr1* knockout cells (red) and $eBFP2^+$ competitor cells (blue) was measured in myeloid, B and T cells in the peripheral blood on week 18 after transplantation. (**g**) Bone marrow (BM), spleen (Spl) and thymus (Thy) were analyzed for the presence of *Egr1* knockout cells (red) and competitor cells (blue) 18 weeks after transplantation. (**h**) The lineage distribution in the bone marrow was analyzed 18 weeks after transplantation by measuring $CD11b^+$ myeloid cells, $B220^+$ B cells and $CD3^+$ T cells. (**i**) Percentages of $dTomato^+$ *Egr1* knockout cells (red) and $eBFP2^+$ competitor cells (blue) were assessed in myeloid, B and T cells in the bone marrow of transplanted animals on week 18 post-transplantation. (**j**) The frequency of $Sca1^+$ $c\text{-}Kit^+$ $Lineage^-$ cells (LSK) was measured in the bone marrow 18 weeks after transplantation and compared to untransplanted age-matched control animals (circles). (**k**) On week 18 post-transplantation, the frequency of $dTomato^+$ *Egr1* knockout cells (red) and $eBFP2^+$ competitor cells (blue) were analyzed in LSK cells in the bone marrow of transplanted mice. (**l**) The white blood cell count (WBC) was quantified in the peripheral blood in mice transplanted with *Egr1* knockout cells on week 18 after transplantation and compared to age-matched untransplanted control animals (circles). Statistics: n=11, mean ± SD, two independent experiments, t-test (b, j-l), two-way ANOVA comparing row means (d, f, g, i) or column means only (e, h), ** $p<0.01$, **** $p<0.0001$.

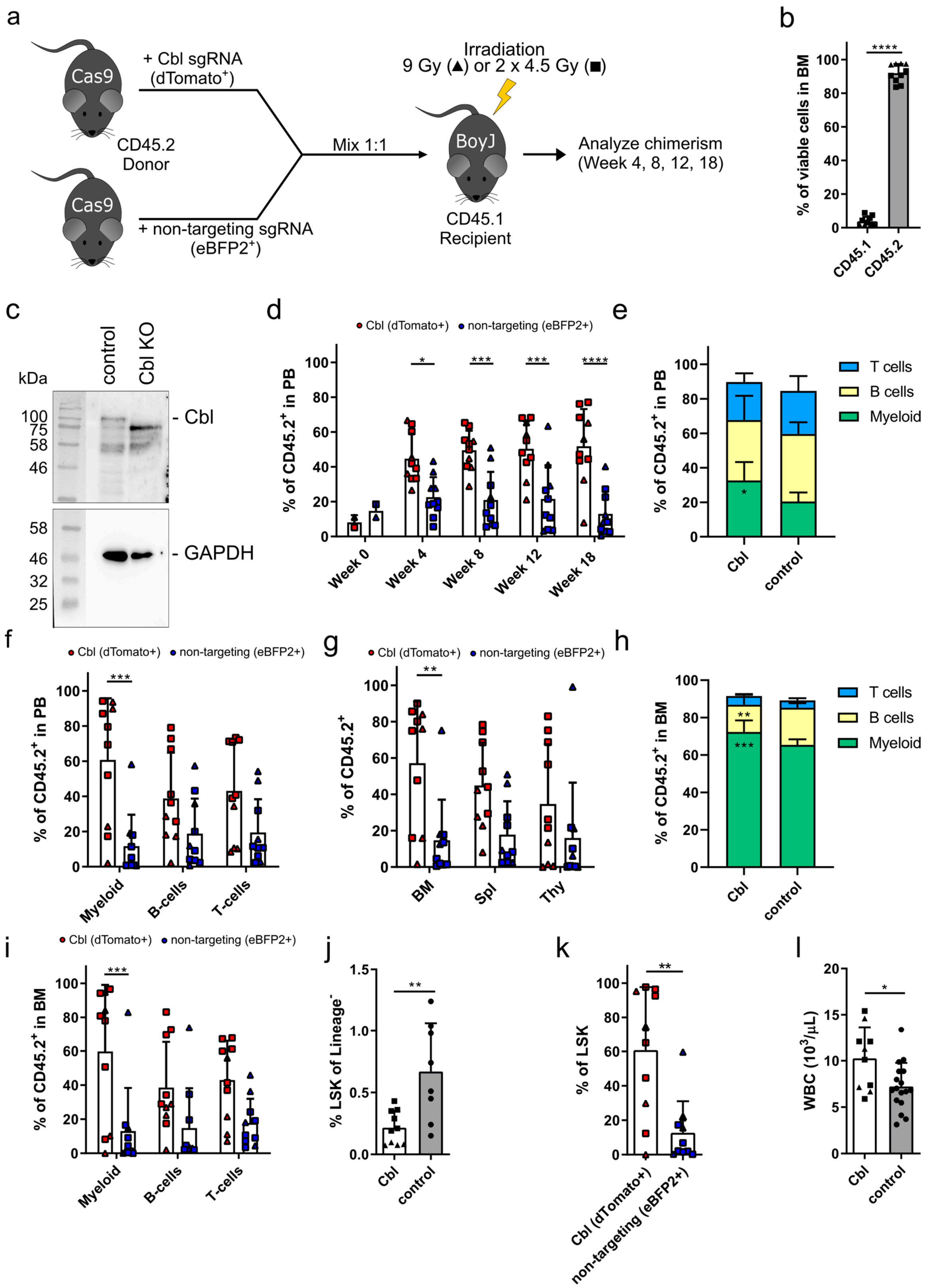

a
Cas9
+ Cbl sgRNA (dTomato+)
CD45.2 Donor
+ non-targeting sgRNA (eBFP2+)
Mix 1:1
Irradiation 9 Gy (▲) or 2 x 4.5 Gy (■)
BoyJ
CD45.1 Recipient
Analyze chimerism (Week 4, 8, 12, 18)
b
% of viable cells in BM
CD45.1
CD45.2
c
kDa
control
Cbl KO
- Cbl
- GAPDH
d
Cbl (dTomato+)
non-targeting (eBFP2+)
% of CD45.2+ in PB
Week 0
Week 4
Week 8
Week 12
Week 18
e
% of CD45.2+ in PB
T cells
B cells
Myeloid
Cbl
control
f
% of CD45.2+ in PB
Myeloid
B-cells
T-cells
g
% of CD45.2+
BM
Spl
Thy
h
% of CD45.2+ in BM
i
% of CD45.2+ in BM
j
% LSK of Lineage-
k
% of LSK
Cbl (dTomato+)
non-targeting (eBFP2+)
l
WBC (10³/µL)

Supplement Figure S3. CRISPR-Cas9-mediated knockout of *Cbl* increases the engraftment of HSPCs in a competitive bone marrow transplantation. (**a**) Schemata of the competitive bone marrow transplantation. Using lentiviral vectors, *Cbl* was knocked out in CD45.2$^+$ HSPCs from Cas9 mice (labeled with a dTomato fluorescent reporter) and transplanted into irradiated CD45.1 mice. As competitors, HSPCs were transduced with a non-targeting sgRNA and an eBFP2 fluorescent reporter. Mice were irradiated with either a single dose of 9 Gy (triangles) or a fractionated dose of 2x 4.5Gy (squares). Peripheral blood was analyzed 4, 8 and 12 weeks after transplantation. On week 18, the animals were euthanized for final analysis. (**b**) The donor cell chimerism was analyzed as CD45.1$^+$ and CD45.2$^+$ cells in the bone marrow 18 weeks after transplantation. (**c**) The knockout of *Cbl* was verified by western blot analysis of a representative bone marrow sample. The expected 126 kDa band for CBL was clearly observed in the control cells and absent from the *Cbl* knockout cells. GAPDH was used as loading control. KO=knockout. (**d**) The frequency of *Cbl* knockout HSPCs (red) and competitor cells (blue) was monitored over time in the peripheral blood of transplanted animals. (**e**) The lineage distribution for myeloid (CD11b$^+$), B (B220$^+$) and T (CD3$^+$) cells was assessed in the peripheral blood of transplanted animals 18 weeks after transplantation in comparison to age-matched control animals. (**f**) The frequency of dTomato$^+$ *Cbl* knockout cells (red) and eBFP2$^+$ competitor cells (blue) was measured in myeloid, B and T cells in the peripheral blood on week 18 after transplantation. (**g**) Bone marrow (BM), spleen (Spl) and thymus (Thy) were analyzed for the presence of *Cbl* knockout cells (red) and competitor cells (blue) 18 weeks after transplantation. (**h**) The lineage distribution in the bone marrow was analyzed 18 weeks after transplantation by measuring CD11b$^+$ myeloid cells, B220$^+$ B cells and CD3$^+$ T cells. (**i**) The presence of dTomato$^+$ *Cbl* knockout cells and eBFP2$^+$ competitor cells were assessed in myeloid, B and T cells in the bone marrow of transplanted animals on week 18 post-transplantation. (**j**) The frequency of Sca1$^+$ c-Kit$^+$ Lineage$^-$ cells (LSK) was measured in the bone marrow 18 weeks after transplantation and compared to untransplanted age-matched control mice (circles). (**k**) On week 18 post-transplantation, the frequency of dTomato$^+$ *Cbl* knockout cells (red) and eBFP2$^+$ competitor cells (blue) were analyzed in LSK cells in the bone marrow of transplanted mice. (**l**) The white blood cell count (WBC) was quantified in the peripheral blood in mice transplanted with *Cbl* knockout cells on week 18 after transplantation and compared to age-matched untransplanted control animals (circles). Statistics: n=11, mean ± SD, two independent experiments, t-test (b, j-l), one-way ANOVA comparing row means (d, f, g, i) or column means only (e, h), * $p<0.05$, ** $p<0.01$, *** $p<0.001$, **** $p<0.0001$.

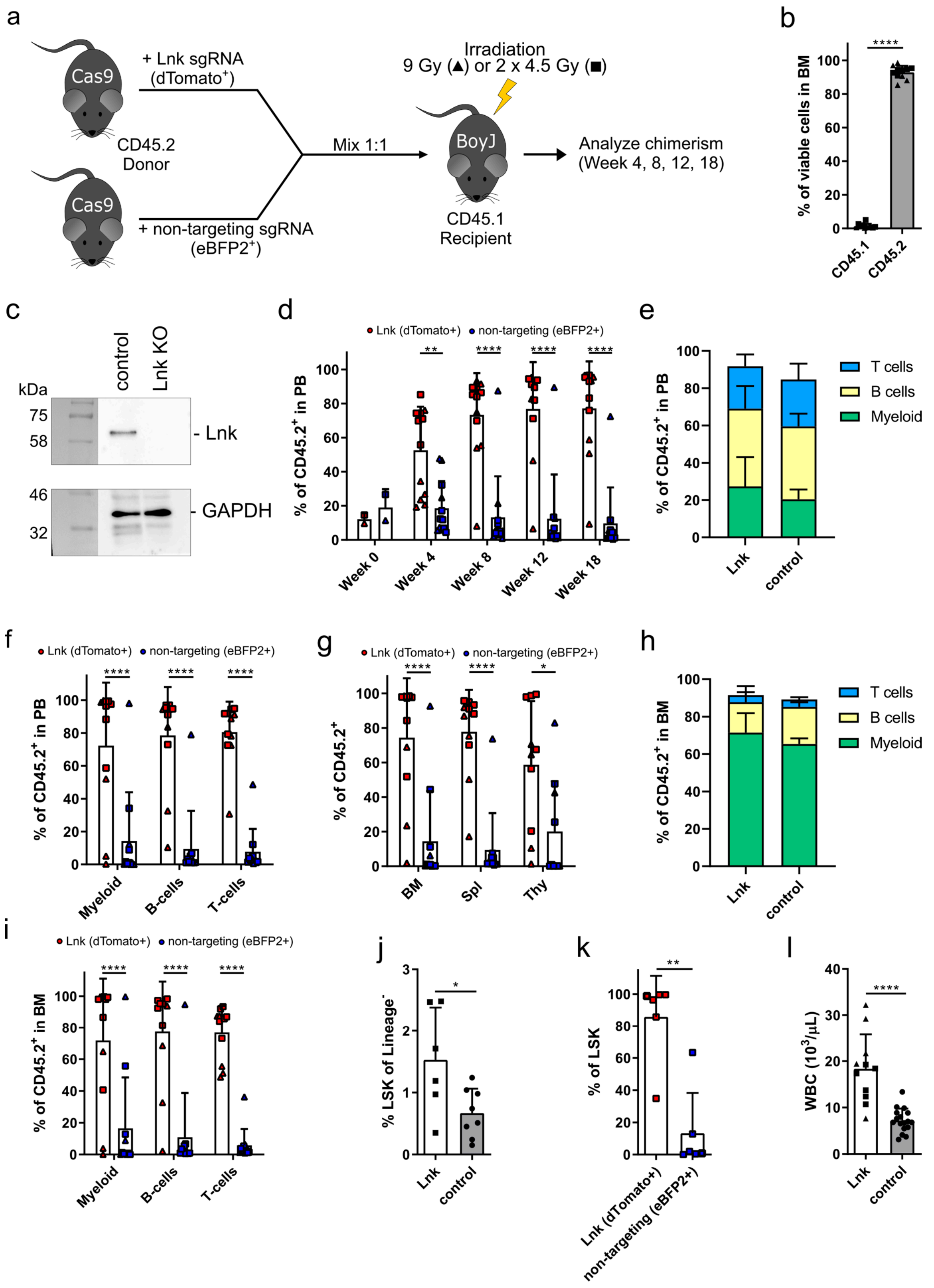
a
+ Lnk sgRNA
(dTomato+)
Cas9
CD45.2
Donor
Cas9
+ non-targeting sgRNA
(eBFP2+)
Irradiation
9 Gy (▲) or 2 x 4.5 Gy (■)
Mix 1:1
BoyJ
CD45.1
Recipient
Analyze chimerism
(Week 4, 8, 12, 18)
b
% of viable cells in BM
CD45.1
CD45.2
c
kDa
control
Lnk KO
- Lnk
- GAPDH
d
Lnk (dTomato+)
non-targeting (eBFP2+)
% of CD45.2+ in PB
Week 0
Week 4
Week 8
Week 12
Week 18
e
T cells
B cells
Myeloid
Lnk
control
f
Myeloid
B-cells
T-cells
g
% of CD45.2+
BM
Spl
Thy
h
% of CD45.2+ in BM
i
j
% LSK of Lineage-
k
% of LSK
Lnk (dTomato+)
non-targeting (eBFP2+)
l
WBC (10³/µL)

Supplement Figure S4. CRISPR-Cas9-mediated knockout of *Lnk* significantly increases the engraftment of HSPCs in a competitive bone marrow transplantation. (**a**) Schemata of the competitive bone marrow transplantation. Using lentiviral vectors, *Lnk* was knocked out in CD45.2$^+$ HSPCs from Cas9 mice (labeled with a dTomato fluorescent reporter) and transplanted into irradiated CD45.1 mice. As competitors, HSPCs were transduced with a non-targeting sgRNA and an eBFP2 fluorescent reporter. Mice were irradiated with either a single dose of 9 Gy (triangles) or a fractionated dose of 2x 4.5 Gy (squares). Peripheral blood was analyzed 4, 8 and 12 weeks after transplantation. On week 18, the animals were euthanized for final analysis. (**b**) The donor cell chimerism was analyzed as CD45.1$^+$ and CD45.2$^+$ cells in the bone marrow 18 weeks after transplantation. (**c**) The knockout of *Lnk* was verified by western blot analysis of a representative bone marrow sample. GAPDH was used as loading control. KO=knockout. (**d**) The frequency of *Lnk* knockout HSPCs (red) and competitor cells (blue) was monitored over time in the peripheral blood of transplanted animals. (**e**) The lineage distribution for myeloid (CD11b$^+$), B (B220$^+$) and T (CD3$^+$) cells was assessed in the peripheral blood of transplanted animals 18 weeks after transplantation in comparison to age-matched control animals. (**f**) The frequency of dTomato$^+$ *Lnk* knockout cells (red) and eBFP2$^+$ competitor cells (blue) was measured in myeloid, B and T cells in the peripheral blood on week 18 after transplantation. (**g**) Bone marrow (BM), spleen (Spl) and thymus (Thy) were analyzed for the presence of *Lnk* knockout cells (red) and competitor cells (blue) 18 weeks after transplantation. (**h**) The lineage distribution in the bone marrow was analyzed 18 weeks after transplantation by measuring CD11b$^+$ myeloid cells, B220$^+$ B cells and CD3$^+$ T cells. (**i**) The presence of dTomato$^+$ *Lnk* knockout cells and eBFP2$^+$ competitor cells were assessed in myeloid, B and T cells in the bone marrow of transplanted animals on week 18 post-transplantation. (**j**) The frequency of Sca1$^+$ c-Kit$^+$ Lineage$^-$ cells (LSK) was measured in the bone marrow 18 weeks after transplantation and compared to untransplanted, age-matched control mice (circles). (**k**) On week 18 post-transplantation, the frequencies of dTomato$^+$ *Lnk* knockout cells (red) and eBFP2$^+$ competitor cells (blue) were analyzed in LSK cells in the bone marrow of transplanted mice. (**l**) The white blood cell count (WBC) was quantified in the peripheral blood in mice transplanted with *Lnk* knockout cells on week 18 after transplantation and compared to age-matched untransplanted control animals (circles). Statistics: n=11, mean ± SD, two independent experiments, t-test (b, j-l), one-way ANOVA comparing row means (d, f, g, i) or column means only (e, h), * $p<0.05$, ** $p<0.01$, **** $p<0.0001$.

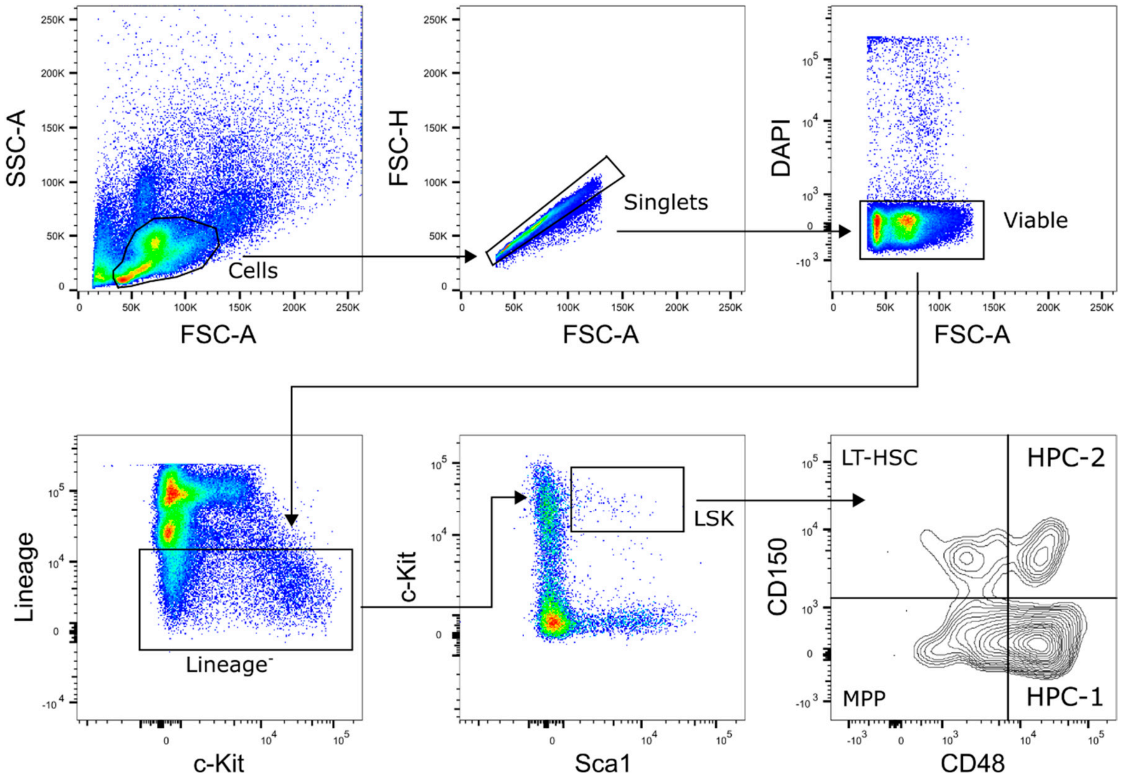

Supplement Figure S5. Gating strategy for HSPC subpopulations.

Supplementary Reference

1. Stemmer, M.; Thumberger, T.; Del Sol Keyer, M.; Wittbrodt, J.; Mateo, J.L. CCTop: An Intuitive, Flexible and Reliable CRISPR/Cas9 Target Prediction Tool. *PLoS ONE* **2015**, *10*, e0124633, doi:10.1371/journal.pone.0124633.
